# State of Knowledge and Data Gaps of Middle East Respiratory Syndrome Coronavirus (MERS-CoV) in Humans

**DOI:** 10.1371/currents.outbreaks.0bf719e352e7478f8ad85fa30127ddb8

**Published:** 2013-11-12

**Authors:** The WHO MERS-CoV Research Group, Mohammad Mousa Abdallat, Fekri Abroug, Said Hamed Al-Dhahry, Mohammed M. Al-Hajri, Rafat Al-Hakeem, Farida Ismail AlHosani, Sultan Mohammed Abdalla Al Qasrawi, Hamad Eid Al-Romaihi, Abdullah Assiri, J Kenneth Baillie, Peter Karim Ben Embarek, Afif Ben Salah, Benjamin Blümel, Thomas Briese, Udo Buchholz, Sebastien Bruno Francois Cognat, Gabriel N. Defang, Stéphane De La Rocque, Isabella Donatelli, Christian Drosten, Patrick A. Drury, Sergey R. Eremin, Neil M. Ferguson, Arnaud Fontanet, Pierre B.H. Formenty, Ron A.M. Fouchier, Christine Q Gao, Erika Garcia, Susan Gerber, Benoît Guery, Bart L. Haagmans, Aktham Jeries Haddadin, Maxwell C. Hardiman, Lisa E. Hensley, Stéphane Hugonnet, David SC Hui, Nicolas Isla, William Karesh, Marion Koopmans, Anna Kuehne, W. Ian Lipkin, Ali R. Mafi, Mamunur Malik, Jean-Claude Manuguerra, Ziad Memish, Anthony W. Mounts, Elizabeth Mumford, Langoya Opoka, Ab Osterhaus, Christopher J. Oxenford, Vincent Pang, Richard Pebody, J. S. Malik Peiris, Bruce Jay Plotkin, Gilles Poumerol, Chantal Reusken, Giovanni Rezza, Cathy E. Roth, Nahoko Shindo, Alice M. Shumate, Molly A. Siwula, Amine Slim, Catherine Smallwood, Sylvie van der Werf, Maria D. Van Kerkhove, Maria Zambon

## Abstract

Background: Between September 2012 and 22 October 2013, 144 laboratory-confirmed and 17 probable MERS-CoV cases from nine countries were notified to WHO. Methods: We summarize what is known about the epidemiology, virology, phylogeny and emergence of MERS-CoV to inform public health policies. Results: The median age of patients (n=161) was 50 years (range 14 months to 94 years), 64.5% were male and 63.4% experienced severe respiratory disease. 76.0% of patients were reported to have ≥1 underlying medical condition and fatal cases, compared to recovered or asymptomatic cases were more likely to have an underlying condition (86.8% vs. 42.4%, p<0.001). Analysis of genetic sequence data suggests multiple independent introductions into human populations and modelled estimates using epidemiologic and genetic data suggest R<sub>0</sub> is <1, though the upper range of estimates may exceed 1. Index/sporadic cases (cases with no epidemiologic-link to other cases) were more likely to be older (median 59.0 years vs. 43.0 years, p<0.001) compared to secondary cases, although these proportions have declined over time. 80.9% vs. 67.2% of index/sporadic and secondary cases, respectively, reported ≥1 underlying condition. Clinical presentation ranges from asymptomatic to severe pneumonia with acute respiratory distress syndrome and multi-organ failure. Nearly all symptomatic patients presented with respiratory symptoms and 1/3 of patients also had gastrointestinal symptoms. Conclusions: Sustained human-to-human transmission of MERS-CoV has not been observed. Outbreaks have been extinguished without overly aggressive isolation and quarantine suggesting that transmission of virus may be stopped with implementation of appropriate infection control measures.

## Background

The first case of a novel coronavirus, now called Middle East respiratory syndrome coronavirus (MERS-CoV), was identified in a patient with acute pneumonia and renal failure in Jeddah, Kingdom of Saudi Arabia (KSA) in June 2012 1. Following identification of the virus a subsequent case from Qatar who was being treated in the UK was identified 2 and a hospital cluster of pneumonia among health care workers in Zarqa, Jordan in March/April of 2012 was retrospectively determined to have been caused by the same virus 3. Since then, as of 22 October 2013, 144 laboratory-confirmed and 17 probable MERS-CoV cases from nine countries (Figure 1a) have been notified to the World Health Organization (WHO) 3 ^,^ 4 ^,^ 5 ^,^ 6 in accordance with the provisions of the International Health Regulations (2005).

WHO, in coordination and cooperation with its affected member states, and an informal network of academic and public health researchers, has compiled available information on MERS-CoV. Here we summarize what is currently known about the epidemiology of MERS-CoV, including the geographic spread and timeline of cases, characteristics and severity of cases, clinical features and treatment strategies, description of clusters, and the epidemic potential of MERS-CoV; and the virology, phylogeny and emergence of the MERS-CoV virus. In addition to summarizing the published literature, we also present summary data on the known laboratory-confirmed and probable cases of MERS-CoV infection that have been reported to WHO. This compilation represents our current state of knowledge of MERS-CoV and the disease it causes and summarizes information needed to inform public health policies for surveillance, preparedness, and response.

**a) Country of probable exposure of laboratory-confirmed MERS-CoV cases b) Number of laboratory confirmed MERS-CoV cases by country of probable exposure:**
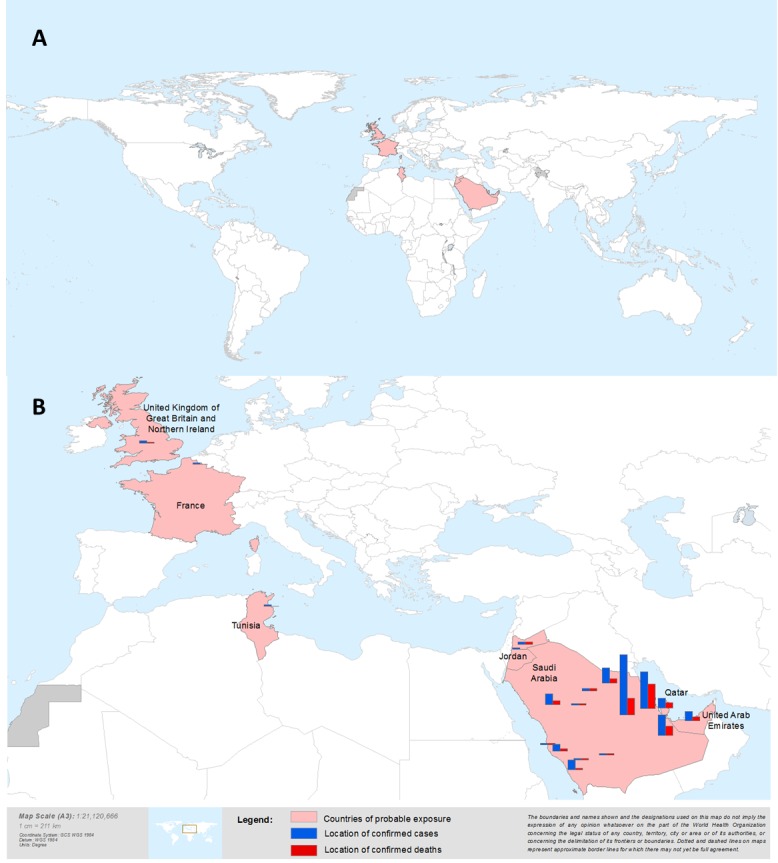
Bars do not represent the exact location of cases. The most detailed level of information for place was mapped when possible. For cases where only the province name was reported, the geographic centre of that province is mapped.

## Epidemiology of MERS-CoV infection in humans

Our understanding of the epidemiology and clinical presentation of MERS-CoV infection is heavily influenced by the recommended surveillance strategies for case detection, which largely focus on severe illness and virologic testing 4, and published detailed investigations of cases. Contact tracing activities allow for the detection of confirmed cases with a broader spectrum of illness. Confirmed cases include only those with a positive polymerase chain reaction (PCR) in accordance with the laboratory guidelines for virus genetic material 7. Probable cases are those that have a link with a confirmed case and a clinically compatible illness but without definitive laboratory confirmation. Antibody testing is not currently being used for confirmatory testing. In addition to summarizing data related to both confirmed and probable cases, we also examined the differences between secondary cases discovered around sporadic cases as a result of contact tracing. While the majority of cases now reported have likely acquired infection through human-to-human transmission the primary sporadic cases in clusters are more likely to have been acquired through contact with non-human sources of the virus. The differences between them may provide clues as to relevant exposures that result in infection.

**Characteristics of MERS-CoV Cases**

Between April 2012 and 22 October 2013, 144 laboratory-confirmed MERS-CoV cases have been identified in nine countries: France, Germany, Italy, Jordan, KSA, Qatar, Tunisia, United Arab Emirates (UAE), and the United Kingdom (UK) (Figure 1a). All cases have had a direct or indirect link with the Middle East. In KSA (Figure 1b), from which more than 80% of laboratory-confirmed cases have been reported, cases have been reported from six of its 13 provinces (Al Qasim, Al Madinah, Ar Riyad, Asir, Ash Sharaqiyah and Makkah)^6^^,^ 8 ^,^ 9. In addition, 17 probable cases have been identified in Jordan (n=11), KSA (n=4) and Italy (n=2) 3 ^,^ 5 ^,^ 6 ^,^ 10 and are included in our analyses. The number of cases reported rose markedly starting in April 2013 compared with the previous six months since virus discovery, with 21 laboratory-confirmed cases reported to have symptom onset in April, 22 in May, 22 cases in June, 14 cases in July, 20 cases in August, 28 cases in September, and 4 cases in October 2013 (Figure 2)

**Epidemiologic Curve of MERS-CoV Confirmed (n=144) and Probable Cases (n=17):**
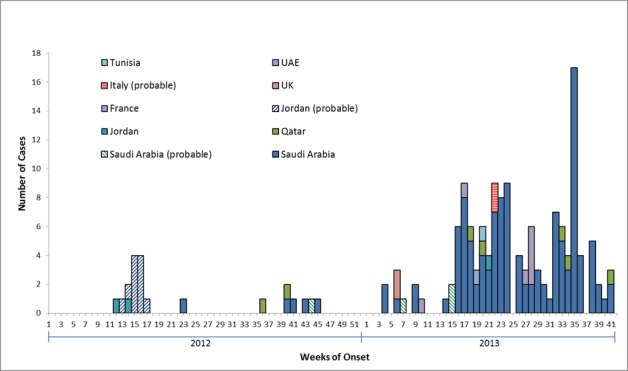
*After week 24 in 2013, 58 cases were not reported with date for symptom onset. For these 58 cases, the symptom onset date was estimated (date of reporting to WHO minus median of difference between onset date and reporting date of those cases that had both of these dates available; the median difference was calculated by country). Cases are reported by the location where infection is believed to have occurred. Figure includes cases reported as of 22 October 2013.

Overall, the median age of MERS-CoV patients is 50 years (range 14 months to 94 years) and 64.5% of patients were male (Table 1). As of 22 October 2013, WHO has confirmation that 65 patients (40.4%), of whom 62 were confirmed and 3 probable, have died, of these 4 deaths could not be matched up to specific cases due to a lack of identifying information. Outcome is unknown for 45 patients, either because they are still in hospital at the time of writing, their outcome has not been reported, or we are unable to match a fatal case to our line list (n=4). Age and sex varied by outcome, with a higher median age and proportion of male sex among patients who died (58 years old and 80.0%, respectively) versus those who recovered or were asymptomatic (34 years old and 60.0%, respectively).

The majority (63.4%) of patients experienced severe respiratory disease while 29.8% were reported to have non-severe disease, including 18 cases reported as asymptomatic. Severe disease was defined as admission to an intensive care unit [ICU]; use of extracorporeal membrane oxygenation (ECMO), mechanical ventilation, or vasopressors; or reported by the member state as “critical” or “severe”; or who died. Severity is unknown for 6.8% of patients. Fifty-three of the 114 hospitalized patients, for whom we had data, died.

Seventy-six per cent of patients are reported to have at least one underlying medical condition and fatal cases were more likely to have an underlying condition (86.8% among fatal cases vs. 42.4% among recovered or asymptomatic cases, p<0.001, Table 1). Of those who were reported to have at least one comorbid condition, specific comorbid diagnoses were not reported for 47.8% of patients. However, of those with data, the most commonly reported conditions include chronic renal failure (13.3%), diabetes (10.0%), and heart disease (7.5%). Of five patients who were immunocompromised, two reported use of immunosuppressive medication, two reported multiple myeloma, and solid tissue cancer was reported in one patient. A published analysis of 47 confirmed patients from KSA, most of whom were part of the Al Hasa cluster, found that almost all (45/47) had at least one underlying condition, including diabetes, hypertension, chronic cardiac disease and chronic renal disease 9.

We classified 51 patients as likely being sporadic (n=29) or “index” cases (n=22) based on the following criteria: 1) a report of having no exposure to other known cases, 2) occurring in an area with no previous cases or no cases within the last 2 months, or 3) reported as the index case in a cluster or being the first case reported with symptoms in a cluster. Ninety-five cases were classified as secondary cases with epidemiologic links to other confirmed MERS-CoV cases (Table 1). For 17 cases, epidemiological classifications remain unclear as no information about contact with other cases is available.

Seventy-three per cent of index/sporadic cases were male, as compared with 60.0% of secondary cases, though this difference was not significant. Notably, this proportion has declined over time; more than 90% of the earliest cases reported were male. Index/sporadic cases were older (median age 59 years vs. 43 years, p<0.001), and more likely to experience severe disease requiring hospitalization or advanced care (90.2% vs. 49.5%, p<0.001) (Table 1). A similar proportion of index/sporadic cases and secondary cases reported at least one underlying condition (80.9% and 67.2%, respectively), and diabetes, heart disease and immunosuppression were most often reported among index/sporadic cases.

The majority (90.2%) of index/sporadic cases had severe or fatal disease (Table 1). However, the proportion with reported chronic renal failure was higher among secondary cases, which is related to the large outbreak related to haemodialysis units in hospitals in Al Hasa 10. Only one of the secondary cases (n=95) that occurred outside of the Al Hasa outbreak reported chronic renal failure.

Among secondary cases, 13 MERS-CoV cases are believed to have been infected in household settings, 60 in health care settings (HCS), and one in a workplace other than a HCS. For twenty-one cases, the place of transmission was not reported. Cases associated with HCS in Jordan, France, KSA, UK, UAE, and Qatar 3 ^,^ 10 ^,^ 11 ^,^ 12 included HCW (30 cases) treating MERS-CoV patients, patients seeking treatment in hospitals for conditions unrelated to MERS-CoV (19 cases) and visitors (6 cases). The type of exposure of five additional secondary cases associated with HCS is unknown. In households and the non-HCS workplace, secondary cases occurred among family contacts or co-workers. The specific types of exposure resulting in transmission are currently unknown.

Only 49 cases have information on exposure to animals, including owning or visiting a farm with camels, goats, sheep, chickens, ducks or other animals. Of these, exposure to animals has been reported for only a few cases (n=7; Table 1).

**Figure:**
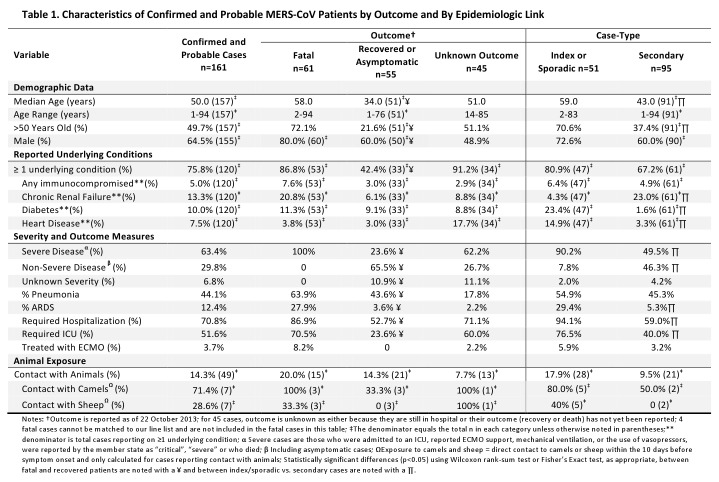

**Clinical Features of MERS-CoV infection in Humans**

Data suggest that the clinical presentation of MERS-CoV infection ranges from asymptomatic to very severe pneumonia with the acute respiratory distress syndrome (ARDS), septic shock and multi-organ failure resulting in death. At least two cases had a consumptive coagulopathy during the course of their illness. The clinical course is more severe in patients with immunocompromising conditions and more likely to be mild in individuals without underlying medical conditions (Table 1). Only three cases have been reported in children under 5 years of age 4.

Typically, the disease starts with fever and cough 3 ^,^ 9 ^,^ 10 ^,^ 11, chills, sore throat, myalgia and arthralgia^2^^,^ 5 ^,^ 9 ^,^ 11, followed by dyspnoea 5 ^,^ 9 ^,^ 10, and rapidly progresses to pneumonia, often requiring ventilatory and other organ support. Nearly all symptomatic patients presented with respiratory symptoms; however, one immunocompromised patient was initially admitted to hospital with fever, chills and diarrhoea and later found to have pneumonia [11]. At least one-third of patients also had gastrointestinal symptoms, such as vomiting and diarrhoea 5 ^,^ 9 ^,^ 10 ^,^ 11. Almost half of the patients developed pneumonia (44.1%), and 20 (12.4%) developed ARDS (Table 1).

Chest radiograph findings vary but are consistent with viral pneumonitis and ARDS: bilateral hilar infiltration, uni- or bilateral patchy densities or infiltrates, segmented or lobar opacities, ground glass appearance, and small pleural effusions have been described. Lower lobes tend to be affected more than upper lobes early in the course of illness; radiographic appearance progresses rapidly. Computed tomographic scans have shown interstitial infiltrates and consolidation compatible with ARDS in severe cases. In some severe cases, renal failure developed concurrently with respiratory failure.

Common laboratory findings include leucopoenia, particularly lymphopaenia 1 ^,^ 3 ^,^ 5 ^,^ 11. Reports from a few cases found viral RNA in blood 11, urine 13 and stool 13 but at much lower viral loads than in the respiratory tract. The viral load in upper respiratory tract specimens is general lower than in the lower respiratory specimens, though data are limited. Co-infection with other respiratory viruses (e.g., parainfluenza, rhinovirus, influenza A(H1N1)pdm09, herpes simplex, influenza B) has been reported in some patients and secondary nosocomially acquired bacterial infections (*Klebsiella pneumoniae, Staphylococcus aureus, Acinetobacter sp., Candida sp*.) have been reported in patients who received mechanical ventilation 1 ^,^ 2 ^,^ 12 ^,^ 13.

The median times from symptom onset to hospitalization, admission to an ICU or to death are 4.0 days (range 0-16, n=62), 5.0 (1-15, n=35) and 11.5 days (4-298, n=40), respectively. The duration of hospitalization to either discharge or death was relatively short, with a median of 7.0 days (2-39, n=21) and 9.0 (2-293, n=24) days, respectively. One patient, who died after 298 days of symptom onset, had been treated with ECMO from the third week of illness 2. Two additional cases died after 35 days or more in hospital, having undergone prolonged ECMO support.

**Treatment**

In the absence of pathogen-specific interventions, patient management largely depends on provision of organ support, and vigilance for and prevention of complications. In specific circumstances, additional interventions have included empiric use of broad-spectrum antimicrobial agents, antivirals (oseltamivir and/or acyclovir), and the addition of anti-fungal agents to minimize risk of co-infections with opportunistic pathogens.

Lung-protective ventilatory strategies for ARDS, cardiovascular support, antimicrobial therapy for co-infections, and renal replacement therapy for acute renal failure have been used 1 ^,^ 2 ^,^ 11 ^,^ 13. Case reports of patients supported with ECMO are available for six patients, five of whom have died 2 ^,^ 11 ^,^ 12. No case-control data exist to evaluate the effectiveness of such interventions.

Many patients with severe disease have been treated with systemic high-dose corticosteroids, which was intended to reverse the progression of respiratory distress and to prevent lung fibrosis. This appears to have been unsuccessful.

None of the antimicrobial agents used so far, including the antivirals, appear to be successful in improving severe progressive disease. A large number of pharmacological agents have been screened against MERS-CoV and several agents have shown inhibitory effects against MERS-CoV in cell cultures. Among the agents tested in vitro are interferons, cyclosporin A, ribavirin, nitazoxanide, immunoglobulins, lopinavir and SARS CoV convalescent plasma^14^^,^ 15 ^,^ 16 ^,^ 17 ^,^ 18. Currently, no clinical data support use of these agents. However, a recent study has found favorable outcomes in MERS-CoV infected rhesus macaques treated with ribavirin and interferon-alpha 2b65.

## Transmission of MERS-CoV

**Clusters ^[1]^**

_*[1] WHO defines a “cluster” as two or more persons with onset of symptoms within the same 14 day period, and who are associated with a specific setting, such as a classroom, workplace, household, extended family, hospital, other residential institution, military barracks or recreational camp (WHO Interim Surveillance Recommendations, 2013).*_

A number of the cases in France, Italy, Jordan, KSA, Tunisia, UAE, UK and Qatar have been reported in clusters 1 ^,^ 4, providing evidence that human-to-human transmission has occurred in HCS, households, and the workplace (Table 2) 3 ^,^ 5 ^,^ 10 ^,^ 11 ^,^ 12 ^,^ 64 . In France, Italy, Jordan, KSA, Qatar, Tunisia, UAE and UK, secondary cases were identified through intense case-finding and follow-up of contacts. Intensity of follow-up of close contacts of patients varies between countries and has included screening contacts for respiratory symptoms and evidence of infection either using reverse-transcriptase PCR (RT-PCR) or, in some situations, serologic assays (Table 2).

As the exposure that results in sporadic infection is unknown, it is impossible to estimate the incubation period in these cases. However, data for human-to-human transmission in the clusters are available from France 11 ^,^ 19, UK 12, Italy 20, Tunisia 21, KSA^10^ and Jordan. The incubation period has been estimated to be just over five days, but could be as long as two weeks (median 5.2 days (95% CI: 1.9 to 14.7)^10^; 5.5 (95% CI: 3.6-10.2)^22^). The incubation period for the primary cases who acquired infection from environmental or animal sources is unknown.

Transmission in all reported clusters has been observed to be limited, and current evidence from contact tracing suggests that transmission did not extend beyond close contacts into the community. Among clusters around exported cases travelling to France, UK, Italy, Germany and Tunisia from the Middle East, transmission to close contacts has been limited 6 ^,^ 11 ^,^ 12 ^,^ 20 ^,^ 23, and secondary attack rates among family members of patients in other clusters appear to be low^3^^,^ 5 ^,^ 6 ^,^ 10 ^,^ 12 ^,^ 24 ^,^ 25 . Systematic implementation of infection prevention and control measures in reported clusters involving HCS has appeared to limit onward transmission to HCW and hospitalized patients 10 ^,^ 11 ^,^ 12.

In HCWs infected by exposure to patients, fewer underlying conditions and milder presentations have been described; however, 37.5% experienced severe disease and at least three died. None of the HCW secondary cases had reported underlying immunosuppression; however, two had hypertension and one had an unspecified underlying comorbid condition. Among secondary cases in HCS who were patients admitted to hospital for other conditions at the time of MERS-CoV infection (n=19), all had underlying conditions, 5.3% reported immunosuppressing conditions, all experienced severe disease, and 89.5% died.

**Figure:**
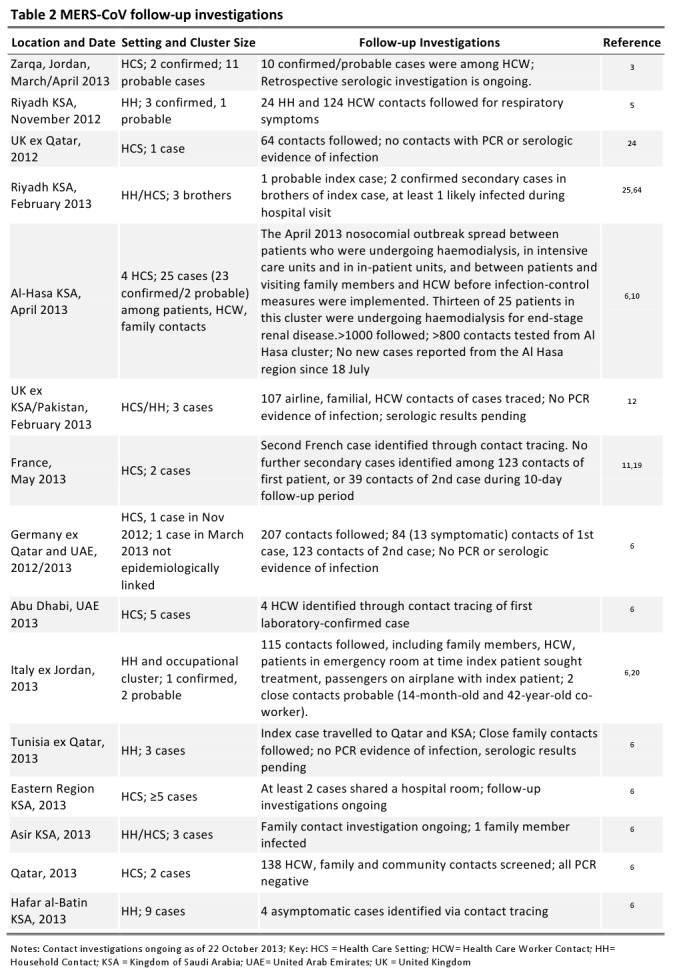

**Epidemic Potential of MERS-CoV**

Two groups have evaluated the transmission potential of the MERS-CoV virus by estimating the basic reproduction number (the number of secondary cases that one case would produce in a completely susceptible population, *R_0_*)^22^^,^ 26.

Breban and colleagues 26 used the first 55 laboratory-confirmed cases of MERS-CoV reported to WHO to assess the inter-human transmissibility of MERS-CoV. The authors estimated *R_0_* from the distribution of sizes of case transmission trees seen so far and compared their estimate with that of early data from the SARS-CoV epidemic in 2003. Two scenarios were considered. With the most pessimistic scenario, the authors estimated MERS-CoV *R_0_* to be 0.69 (95% CI 0.50–0.92); by contrast, the *R_0 _*for SARS-CoV was 0.80 (0.54–1.13). The optimistic scenario resulted in a *R_0_* of 0.60 (0.42–0.80). The authors suggest that, based on their analyses using PCR-confirmed cases, MERS-CoV does not yet have pandemic potential.

Cauchemez and colleagues 22 undertook independent analyses to assess the transmissibility and extent of spread of MERS-CoV to date. Using publically available epidemiological data on 111 confirmed and probable MERS-CoV patients and genetic sequence data from 10 cases, the study found central estimates of R*_0_* between 0.8 and 1.3. This work suggests that current data are consistent with two scenarios: (a) a sustained epidemic in an animal reservoir with sporadic spill-over into humans, or (b) sustained human-to-human transmission causing a slowly growing human epidemic. They also used epidemiological and genetic data to evaluate the underlying scale of the epidemic so far. Analyses using numbers of exported cases of returning travellers from countries in the Middle East (n=4) and average length of visitor stays to Jordan, Qatar, KSA and UAE suggest that by now at least 900 symptomatic cases of MERS-CoV have occurred, implying substantial under-ascertainment of cases in the region. Genetic analyses suggest that approximately 17490 (IQR 3900-95000) infections in humans and in the reservoir(s) may have occurred between June 2012 and August 2013 22.

An analysis of 21 genetic sequences from cases in KSA was compared to those already available in public databases and concluded that the genetic diversity of available sequences supported multiple introductions from a presumed zoonotic source, with subsequent human-to-human transmission^27^. However, the authors could not exclude the possibility of unrecognized sustained transmission in humans associated with frequent movement of infected individuals to explain the observed genetic patterns

**Animal Reservoir**

Several lines of evidence support the hypothesis that the virus originates in animals. Limited phylogenetic analyses using genetic material collected from MERS-CoV patients’ specimens shows that MERS-CoV has a close genetic relationship with coronaviruses found in hedgehogs (Corman et al unpublished) and in bats in Southern China^1^^,^ 28, Europe^29^^,^ 30, Thailand^31^, Mexico^32^,Ghana^30^, and South Africa^33^. A 190-nucleotide fragment of the RNA-dependent RNA polymerase gene was recovered from a faecal sample from a *Taphozous perforatesbat* collected in Bisha, KSA, near the home of the first MERS-CoV patient^34^. The fragment had 100% nucleotide identity with a MERS-CoV recovered from the human case that occurred in that area.

Neutralizing antibodies against MERS-CoV or a similar virus have been described in camels from the Spanish Canary Islands, Oman^35^ and from Egypt^36^. Approximately 15% of sera from camels from the Spanish Canary Islands, 100% of sera from unrelated retired racing camels from Oman and more than 90% of dromedary camels from Egypt were found to have antibody titres reactive with MERS-CoV using a variety of methods, including neutralization. No viral genetic material was detected in a limited number of tested camel sera and faecal samples.

## Virological Characteristics of MERS-CoV

MERS-CoV is an enveloped, single-stranded, positive-sense RNA virus that is a newly recognized species in lineage C of the genus *Betacoronavirus* within the subfamily *Coronavirinae* 1 ^,^ 28. The genome is approximately 30.1kb long and contains at least 10 predicted open reading frames (ORF) common to betacoronaviruses, which are expressed from seven subgenomic mRNAs^28^. These ORFs mainly include ORF 1a/1ab, which encode for large replicase polyproteins containing conserved functional domains and several non-structural (NS) proteins of CoV, the spike-surface glycoprotein (S), the small-envelope (E) protein, the matrix (M) protein, and the nucleocapsid (N) protein.

**Laboratory Testing of MERS-CoV**

Initial detection of viral genome in human clinical samples was made using pancorona primers targeting highly conserved regions of the coronavirus genome. Specific assays for the detection of acute infection with MERS-CoV by real-time RT-PCR (rRT-PCR) have subsequently been developed. Several assays are now in widespread use, including those targeting a region upstream of the E gene (upE) or regions within ORF 1b (nsp14 protein)^37^, ORF 1a (nsp6 protein)^39^, and the nucleocapsid protein gene^38^. The assays for the upE and the ORF 1a targets are considered equally sensitive, while the ORF 1b assay is considered less sensitive than the ORF 1a assay. The upE assay has been recommended for screening specimens with the ORF 1a assay or other specific gene targets being used for confirmation^39^.

Currently, laboratory confirmation of a case is considered as a positive PCR result for at least two different specific targets on the MERS-CoV genome or one positive PCR result for a specific target on the MERS-CoV genome and an additional different PCR product sequenced, confirming identity to known sequences of MERS-CoV^7^. Two suitable targets for sequencing are an RNA-dependent RNA polymerase (RdRp) and nucleocapsid (N) protein genes. Serologic assays for MERS-CoV are under development by several laboratories.

**Genetics and Emergence of MERS-CoV**

Twenty-two whole sequences, and partial genome sequences from 9 additional cases, have been published in GenBank, from thirty different MERS-CoV infected patients (Table 3) 2 ^,^ 3 ^,^ 10 ^,^ 12 ^,^ 13 ^,^ 22 ^,^ 23 ^,^ 27 ^,^ 28 ^,^ 29. Phylogenetic trees have been published by several groups^10^^,^ 13 ^,^ 22 ^,^ 29 and are not shown here.

Several estimates of the time frame of the emergence of MERS-CoV in humans have been developed using a variety of analytical tools with different statistical assumptions, and using both whole-genome sequences and individual genes derived from infections at different time points^10^^,^ 13 ^,^ 22 ^,^ 27 ^,^ 29. All suggest the emergence of MERS-CoV in mid-2011, with the range of possible dates ranging broadly from November 2009–April 2012. One study of 10 full genome sequences, including one from a French patient^22^, suggests the emergence in June 2011 with a narrower timeframe, from Sept 2010–Nov 2011 (Table 3). The study using most available sequences, estimates emergence in July 2011 with a broad credible interval, from July 2007 to June 2012^27^ (Table 3). Future studies providing more genome sequences from humans and animals should add to our understanding of transmission patterns and increase the accuracy of the estimated genomic evolutionary rate.

**Figure:**
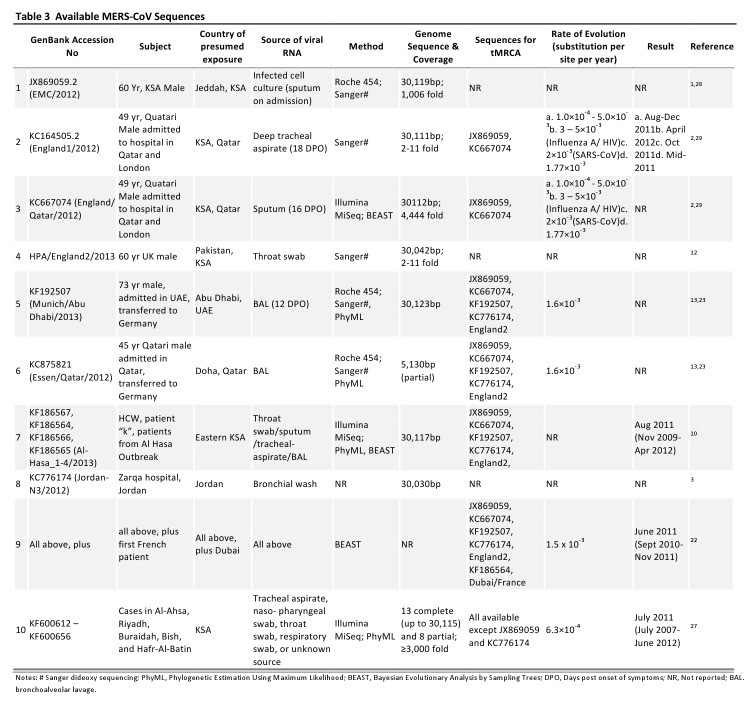

**MERS-CoV - Host Interactions**

The S protein of coronaviruses is responsible for binding to the host cell receptor. The DPP4 (dipeptidyl peptidase 4, also known as CD26)^40^^,^ 41, has been identified as the functional cellular receptor for MERS-CoV, in contrast to the angiotensin-converting enzyme 2 (ACE2), used by the SARS-CoV^42^ and hCoV-NL63. DPP4 homologues permitting MERS-CoV infection are present in a variety of bat, pig, civet and rabbit cell lines, whereas cell lines of canine, feline, rodent, chicken and insect origin were not susceptible^42^^,^ 43.

DPP4 is an exopeptidase and is involved in the regulation of chemokine and cytokine responses and in glucose metabolism^44^. Upon binding, the viral entry of MERS-CoV involves virus-cell fusion, which can be either activated by type II transmembrane serine proteases TMPRSS2 or mediated by low pH and endosomal cathepsins^45^. The receptor-binding domain (RBD) in the S protein responsible for binding to DPP4 has also been mapped. The RBD-S interaction falls within residues 358-662 of the S1 domain, where antibodies to this domain were observed to efficiently neutralize infection^45^^,^ 46 ^,^ 47. Crystal structures of this binding interface showed a homologous core subdomain but much variation in the external receptor binding motif region, compared with that of SARS-CoV^41^.

In humans, DPP4 is primarily expressed on the epithelial cells in kidney, small intestine, liver and prostate, and on activated leukocytes^13^^,^ 40. MERS-CoV has been shown to infect non-ciliated bronchial epithelium, bronchiolar epithelial cells, alveolar epithelial cells, endothelial cells, and lung ex vivo organ cultures at least as well as SARS-CoV^40^^,^ 48 ^,^ 49 ^,^ 50. Receptor preference and underlying tissue tropism may influence the severity of highly pathogenic coronaviruses compared with HCoV-229E infection, which is usually milder and has limited infectivity of these lower respiratory tract cells. However, cellular tropism does not completely account for severity of disease.

Cell-based studies have shown that MERS-CoV, like other coronaviruses, evades innate immune recognition, perhaps accounting for the high proportion of cases observed with severe disease^16^^,^ 18 ^,^ 48 ^,^ 49. However, MERS-CoV was observed to have higher sensitivity to pegylated interferon treatment, compared with SARS-CoV. This has been hypothesized to be due to the lack of a SARS-CoV ORF6 homologue in MERS-CoV, which has been shown to inhibit the nuclear translocation of p-STAT1 and activation of downstream antiviral genes^28^^,^ 51. MERS-CoV was observed to induce a massive dysregulation of the host cellular transcriptome to a much greater extent and in a shorter time after infection than SARS-CoV, resulting in profound apoptosis of surrounding cells^17^^,^ 49 ^,^ 50. This involves the greater suppression of the antigen presentation pathway in MERS-CoV compared with SARS-CoV. This phenomenon suggests that SARS-CoV and MERS-CoV may activate different cellular mechanisms for viral replication and infection, and may delay the adaptive response in the infected host, particularly the immunocompromised patients^17^.

## Discussion and gaps in knowledge

Much has been learned about the MERS-CoV and the disease syndrome it causes in humans since the first case was reported in September 2012, but many critical questions remain unanswered. From the time of the first cases reported to WHO, it was suspected that the virus was of animal origin and subsequent observations since that time have reinforced this view. Current evidence suggests that the virus is most closely related genetically to viruses found in a number of species of old world and new world insectivorous bats^1^^,^ 28 ^,^ 29 ^,^ 30 ^,^ 31 ^,^ 32 ^,^ 33 ^,^ 34. The finding of a gene fragment in a Saudi bat^34^, which is homologous with that of viruses from humans, suggests that bats may be the ultimate reservoir of virus; however, it is very possible that another intermediate animal reservoir could be involved, as was the case with SARS and with Nipah virus in Malaysia. Serological data suggest infections with MERS-CoV or a closely related virus in camels, however the virus itself has not been detected in camels^36^^,^ 35. Currently, all known cases have a direct or indirect link to the Middle East. Although surveillance is more limited in some parts of the world, large clusters like those seen in Al Hasa, KSA and Zarqa, Jordan have not been observed elsewhere. This apparent restriction of transmission from non-human sources to one area of the world may provide clues to the source of exposures. The most likely explanations are either a restriction in the range of the putative host species or exposures related to human behaviours that occur only in the area. Regardless of the reservoir, however, the primary question to be answered is the route of transmission from animal to human. Sporadic and index cases provide the best opportunity for identifying exposures of interest as these are the infections most likely acquired from non-human exposures. The high predominance of older individuals among these cases could be a clue to exposures and demands further investigation using structured case-control studies^53^. The recent increase in numbers of sporadic cases associated with a change in the proportion of male and female cases is concerning. While it could represent changes in surveillance strategies, it may also signal a change in the behaviour of the virus, an expansion of the reservoir, or changes in human exposures.

It is now clear that the virus does transmit from person to person. However, sustained human-to-human transmission has not been observed. The relative ease with which outbreaks have been extinguished without resorting to overly aggressive isolation and quarantine measures and the limited secondary transmission around clusters, suggest that transmission of virus may be readily stopped.

Genetic sequence data indicate that multiple independent introductions into human populations have occurred. In addition, modelled estimates of R_0_ suggest that R_0_ is <1, though the upper range of estimates may slightly exceed 1^22^^,^ 26. Reports of mild and asymptomatic cases discovered through investigations and testing of contacts of confirmed cases, suggest that the focus on severe disease as a surveillance strategy may miss significant numbers of milder or asymptomatic cases^22^. The importance of these milder cases in transmission, however, is uncertain and needs to be assess with seroepidemiologic studies^54^. Evidence from SARS, however, suggests that asymptomatic cases did not transmit infection^55^. It appears likely that the presence of comorbid conditions, particularly when found in high concentrations such as in HCS, can facilitate transmission.

The basic clinical appearance and course of severe cases seen since the earliest reports has not substantially changed; however, it is now clear that milder and even asymptomatic presentations do occur and that the case-fatality rate is lower than observed. The acute renal failure observed in many cases, especially those reported early on, has been primarily associated with hemodynamic compromise, suggesting that it is likely a secondary process. However, receptors for the virus do occur in renal tissue and viral RNA has been detected at low titres in urine. It is important to note that although the primary site of infection has been the respiratory tract, approximately one-third of patients have experienced gastrointestinal symptoms and low titres of virus have been detected in faeces. While generally this is not likely useful for diagnostic purposes, this finding supports current recommendations for adding contact precautions in HCS and may have implications for infection control in the community. The observed differences in proportion of cases that are severe between primary (index/sporadic) and secondary cases is likely a reflection of the surveillance strategy that focuses on severe disease for identifying primary cases and the additional case-finding carried out around them. However, the possibility that the virulence of the virus is different when transmitted from humans than it is when transmitted from non-human sources cannot be excluded based on available evidence. The presence of a pre-existing chronic medical condition likely increases the chance of severe outcome and death.

At this time, treatment for MERS-CoV remains supportive. A number of licensed pharmaceuticals have been tested *in vitro *and testing is currently underway in *vivo*; however, the lack of an appropriate animal model is hampering these efforts. Clinical observation of patients infected with MERS-CoV and experience with other infectious diseases suggest that high-dose corticosteroids are not effective in management of the disease and may be contraindicated for other reasons. Considerable controversy exists about the value of high-dose corticosteroids in the management of ARDS. There is substantial evidence of harm, and no evidence of benefit, from high-dose corticosteroids when used for SARS and influenza A(H1N1)pdm09^4^^,^ 56 ^,^ 57 ^,^ 58 ^,^ 59 ^,^ 60. For this reason, WHO guidance does not recommend the systemic use of high-dose corticosteroids for MERS-CoV treatment, with a few exceptions limited to, for example, patients with asthma or vasopressor refractory shock^61^. It may be that convalescent sera holds some promise until an effective pharmaceutical can be identified. If indeed convalescent sera proves efficacious, specific human monoclonal antibodies may also be considered. Sharing of clinical information and case-management experience is critical to better understand the natural history of disease for MERS-CoV^62^ and improve management guidance. In addition to infection prevention and control measures aiming to minimize transmission of MERS-CoV^63^, attention should be paid to rigorous implementation of safe practices to prevent acquisition of bacterial and fungal pathogens associated with health care.

Many critical questions about MERS-CoV and its related disease remain. First among these is the nature of the exposures that result in human infection. Although an animal reservoir of the virus is the most likely hypothesis, the route of transmission could be either direct or indirect contact with the reservoir or an intermediate host, including consumption of a contaminated food product, or contact with a contaminated fomite. Thus far, spread among humans appears to be relatively easy to extinguish with conventional infection control measures and has not required the aggressive measures that were used to contain SARS. Prevention of transmission from the putative animal reservoir to humans is the critical step needed to halt the ongoing spread of this virus. The answer to how to achieve this and other remaining questions requires formal, structured, multinational collaborative research studies, including the development and implementation of effective surveillance strategies.

## Members of the MERS-CoV Research Group

**Mohammad Mousa Abdallat**

Ministry of Health

Amman, Jordan

**Fekri Abroug**

Intensive Care Department

Fattouma Bourguiba Hospital

Monastir, Tunisia

**Said H.S. Al Dhahry**

Department of Laboratory Medicine

Hamad Medical Corporation

Doha, Qatar

**Mohd Mohd Alhajri**

Supreme Council of Health

Doha, Qatar

**Rafat Al-Hakeem**

Public Health Directorate

Ministry of Health

Riyadh, Saudi Arabia

**Farida Ismail Al Hosani**

Health Authority Abu Dhabi

United Arab Emirates

**Sultan Mohammed Abdalla Al Qasrawi**

Communicable Disease Directorate

Ministry of Health

Amman, Jordan

**Hamad Eid Al-Romaihi**

Supreme Council of Health

Doha, Qatar

**Abdullah Assiri**

Public Health Directorate

Ministry of Health

Riyadh, Saudi Arabia

**J Kenneth Baillie**

The Roslin Institute

University of Edinburgh

Edinburgh, Scotland ****

**Peter Karim Ben Embarek**

Food Safety and Zoonoses

World Health Organization

Geneva, Switzerland

**Afif Ben Salah**

Institut Pasteur de Tunis

Tunis, Tunisia

**Benjamin Blümel** ****

Department of Infectious Disease Epidemiology

Robert Koch Institute

Berlin, Germany

**Thomas Briese**

Center for Infection and Immunity and Department of Epidemiology

Columbia University

Mailman School of Public Health

New York, New York, USA

**Udo Buchholz**

Department of Infectious Disease Epidemiology

Robert Koch Institute

Berlin, Germany

**Sebastien Bruno Francois Cognat**

Support to IHR Capacity Development

World Health Organization

Geneva, Switzerland

**Gabriel N. Defang**

Head, Viral & Zoonotic Diseases Research Program

NAMRU3 - U.S. Naval Medical Research Unit No. 3, Cairo, Arab Republic of Egypt

**Stéphane De La Rocque**

IHR Monitoring, Procedures and Information

World Health Organization

Geneva, Switzerland

**Isabella Donatelli**

National Influenza Centre

Department of Infectious, Parasitic and Immune-Mediated Diseases

Istituto Superiore di Sanità

Roma, Italy

**Christian Drosten**

Institute of Virology

University of Bonn Medical Centre

Bonn, Germany

**Patrick Anthony Drury**

Global Alert and Response Operations

World Health Organization

Geneva, Switzerland

**Sergey Romualdovich Eremin**

AMR, Infection Control and Publications

World Health Organization

Geneva, Switzerland

**Neil M. Ferguson**

MRC Centre for Outbreak Analysis and Modelling

Department of Infectious Disease Epidemiology

Imperial College London

London, United Kingdom

**Arnaud Fontanet**

Emerging Diseases Epidemiology Unit

Institut Pasteur

Paris, France

**Pierre B.H. Formenty**

Control of Epidemic Diseases

World Health Organization

Geneva, Switzerland

**Ron A.M. Fouchier**

Department of Virology

Erasmus MC

Rotterdam, Netherlands

**Christine Qiuhan Gao**

Biodefence Centre

Singapore Armed Forces

Singapore

**Erika Garcia**

Global Alert and Response Operations

World Health Organization

Geneva, Switzerland

**Susan I. Gerber**

Division of Viral Diseases/ Epidemiology Branch

National Center for Immunization and Respiratory Diseases

Centers for Disease Control and Prevention

Atlanta, USA

**Benoît Guery**

Maladies Infectieuses

CHRU

Lille, France

**Bart L. Haagmans**

Viroscience Lab

Erasmus MC

Rotterdam, Netherlands

**Aktham Jeries Haddadin**

National Laboratories Directorate

Ministry of Health

Amman, Jordan

**Maxwell Charles Hardiman**

IHR Monitoring, Procedures and Information

World Health Organization

Geneva, Switzerland

**Lisa E Hensley**

National Institutes of Health

Frederick, Maryland, USA

**Stéphane Alexandre Louis Hugonnet**

Global Alert and Response Operations

World Health Organization

Geneva, Switzerland

**David SC Hui**

The Chinese University of Hong Kong

Department of Medicine & Therapeutics

Prince of Wales Hospital

Hong Kong, SAR

**Nicolas Isla**

Global Alert and Response Operations

World Health Organization

Geneva, Switzerland

**William B. Karesh**

Executive Vice President for Health and Policy

EcoHealth Alliance

New York, New York, USA

**Marion Koopmans**

National Institute of Public Health and the Environment

Bilthoven, Netherlands

**Anna Kuehne**

Department of Infectious Disease Epidemiology

Robert Koch Institute

Berlin, Germany

**W. Ian Lipkin**

Center for Infection and Immunity

Columbia University

New York, New York, USA

**Ali R. Mafi**

Pandemic and Epidemic Diseases

World Health Organization Regional Office for the Eastern Mediterranean

Cairo, Egypt

**Mamunur Malik**

Pandemic and Epidemic Diseases

World Health Organization Regional Office for the Eastern Mediterranean

Cairo, Egypt

**Jean-Claude Manuguerra**

Laboratory for urgent response to biological threats

Institut Pasteur

Paris, France

**Ziad Memish**

Deputy Minister of Health

Ministry of Health

Riyadh, Saudi Arabia

**Anthony Wayne Mounts**

Global Influenza Programme

World Health Organization

Geneva, Switzerland

**Elizabeth Mumford**

Food Safety and Zoonoses

World Health Organization

Geneva, Switzerland

**Langoya Opoka**

****Pandemic and Epidemic Diseases

World Health Organization Regional Office for the Eastern Mediterranean

Cairo, Egypt

**Ab Osterhaus**

Viroscience Lab, Erasmus MC

Rotterdam, Netherlands

**Christopher John Oxenford**

Support to IHR Capacity Development

World Health Organization

Geneva, Switzerland

**Junxiong Pang**

Centre for Infectious Disease Epidemiology and Research

Saw Swee Hock School of Public Health

National University of Singapore

Genome Institute of Singapore

Singapore

**Richard Pebody**

Respiratory Diseases Department

Centre for Infectious Disease Surveillance and Control

Public Health England

London, United Kingdom

**J. S. Malik Peiris**

School of Public Health

The University of Hong Kong

Hong Kong, SAR

**Bruce Jay Plotkin**

IHR Monitoring, Procedures and Information

World Health Organization

Geneva, Switzerland

**Gilles Poumerol**

IHR Monitoring, Procedures and Information

World Health Organization

Geneva, Switzerland

**Chantal Reusken**

National Institute of Public Health and the Environment

Bilthoven, Netherlands

**Giovanni Rezza**

Director, Department of Infectious Diseases

Istituto Superiore di Sanità

Roma, Italy

**Cathy Ellen Roth**

Health Security and the Environment Cluster

Department of Pandemic and Epidemic Diseases

Department of Global Capacities, Alert and Response

World Health Organization

Geneva, Switzerland

**Nahoko Shindo**

AMR, Infection Control and Publications

World Health Organization

Geneva, Switzerland

**Alice M. Shumate**

Centers for Disease Control and Prevention

Atlanta, Georgia, USA

**Molly Siwula**

Global Influenza Programme

World Health Organization

Geneva, Switzerland

**Amine Slim**

National Influenza Center

Charles Nicolle Hospital

Tunis, Tunisia

**Catherine Smallwood**

Global Alert and Response Operations

World Health Organization

Geneva, Switzerland

**Sylvie van der Werf**

Unit of Molecular genetics of RNA viruses

Institut Pasteur, CNRS

University Paris Diderot Sorbonne Paris Cité

Paris, France

**Maria D. Van Kerkhove**

MRC Centre for Outbreak Analysis and Modelling

Department of Infectious Disease Epidemiology

Imperial College London

London, United Kingdom

**Maria Zambon**

Reference Microbiology Services

UK Public Health England

London, England

## Correspondence

Anthony W. Mounts

Global Influenza Programme, World Health Organization, Geneva, Switzerland

Email: mountsa@who.int; Telephone: +41227911062

The opinions expressed in this article are those of the authors and members of the working group and do not necessarily reflect those of the institutions or organizations with which they are affiliated.

## References

[1] Zaki AM, van Boheemen S, Bestebroer TM, Osterhaus AD, Fouchier RA. Isolation of a novel coronavirus from a man with pneumonia in Saudi Arabia. N Engl J Med. 2012 Nov 8;367(19):1814-20. PubMed PMID:23075143. 23075143 10.1056/NEJMoa1211721

[2] Bermingham A, Chand MA, Brown CS, Aarons E, Tong C, Langrish C, Hoschler K, Brown K, Galiano M, Myers R, Pebody RG, Green HK, Boddington NL, Gopal R, Price N, Newsholme W, Drosten C, Fouchier RA, Zambon M. Severe respiratory illness caused by a novel coronavirus, in a patient transferred to the United Kingdom from the Middle East, September 2012. Euro Surveill. 2012 Oct 4;17(40):20290. PubMed PMID:23078800. 23078800

[3] Hijawi B, Abdallat M, Sayaydeh A, Alqasrawi S, Haddadin A, Jaarour N, Alsheikh S, Alsanouri T. Novel coronavirus infections in Jordan, April 2012: epidemiological findings from a retrospective investigation. East Mediterr Health J. 2013;19 Suppl 1:S12-8. PubMed PMID:23888790. 23888790

[4] WHO (2012-2013) World Health Organization. Global Alert and Response. Coronavirus Infections. Available at: http://wwwwhoint/csr/disease/coronavirus_infections/en/

[5] Memish ZA, Zumla AI, Al-Hakeem RF, Al-Rabeeah AA, Stephens GM. Family cluster of Middle East respiratory syndrome coronavirus infections. N Engl J Med. 2013 Jun 27;368(26):2487-94. PubMed PMID:23718156. 23718156 10.1056/NEJMoa1303729

[6] WHO (2012-2013) World Health Organization. Disease Outbreak News Novel Coronavirus. Available at: http://wwwwhoint/csr/don/archive/disease/coronavirus_infections/en/indexhtml Last Accessed 22 October 2013.

[7] World Health Organization (2012) Laboratory testing for novel coronavirus. Interim Recommendations, 21 December 2012. Available at: http://www.who.int/csr/disease/coronavirus_infections/LaboratoryTestingNovelCoronavirus_21Dec12.pdf. Last accessed 22 October 2013.

[8] Saudi Arabia Ministry of Health (2013) Media Statements. Corona. Available at: http://www.moh.gov.sa/en/HealthAwareness/Corona/Pages/PressStatements.aspx, last accessed 22 October 2013.

[9] Assiri A, Al-Tawfiq JA, Al-Rabeeah AA, Al-Rabiah FA, Al-Hajjar S, Al-Barrak A, Flemban H, Al-Nassir WN, Balkhy HH, Al-Hakeem RF, Makhdoom HQ, Zumla AI, Memish ZA. Epidemiological, demographic, and clinical characteristics of 47 cases of Middle East respiratory syndrome coronavirus disease from Saudi Arabia: a descriptive study. Lancet Infect Dis. 2013 Sep;13(9):752-61. PubMed PMID:23891402. 23891402 10.1016/S1473-3099(13)70204-4 PMC7185445

[10] Assiri A, McGeer A, Perl TM, Price CS, Al Rabeeah AA, Cummings DA, Alabdullatif ZN, Assad M, Almulhim A, Makhdoom H, Madani H, Alhakeem R, Al-Tawfiq JA, Cotten M, Watson SJ, Kellam P, Zumla AI, Memish ZA. Hospital outbreak of Middle East respiratory syndrome coronavirus. N Engl J Med. 2013 Aug 1;369(5):407-16. PubMed PMID:23782161. 23782161 10.1056/NEJMoa1306742 PMC4029105

[11] Guery B, Poissy J, el Mansouf L, Séjourné C, Ettahar N, Lemaire X, Vuotto F, Goffard A, Behillil S, Enouf V, Caro V, Mailles A, Che D, Manuguerra JC, Mathieu D, Fontanet A, van der Werf S. Clinical features and viral diagnosis of two cases of infection with Middle East Respiratory Syndrome coronavirus: a report of nosocomial transmission. Lancet. 2013 Jun 29;381(9885):2265-72. PubMed PMID:23727167. 23727167 10.1016/S0140-6736(13)60982-4 PMC7159298

[12] Evidence of person-to-person transmission within a family cluster of novel coronavirus infections, United Kingdom, February 2013. Euro Surveill. 2013 Mar 14;18(11):20427. PubMed PMID:23517868. 23517868 10.2807/ese.18.11.20427-en

[13] Drosten C, Seilmaier M, Corman VM, Hartmann W, Scheible G, Sack S, Guggemos W, Kallies R, Muth D, Junglen S, Müller MA, Haas W, Guberina H, Röhnisch T, Schmid-Wendtner M, Aldabbagh S, Dittmer U, Gold H, Graf P, Bonin F, Rambaut A, Wendtner CM. Clinical features and virological analysis of a case of Middle East respiratory syndrome coronavirus infection. Lancet Infect Dis. 2013 Sep;13(9):745-51. PubMed PMID:23782859. 23782859 10.1016/S1473-3099(13)70154-3 PMC7164791

[14] Chan KH, Chan JF, Tse H, Chen H, Lau CC, Cai JP, Tsang AK, Xiao X, To KK, Lau SK, Woo PC, Zheng BJ, Wang M, Yuen KY. Cross-reactive antibodies in convalescent SARS patients' sera against the emerging novel human coronavirus EMC (2012) by both immunofluorescent and neutralizing antibody tests. J Infect. 2013 Aug;67(2):130-40. PubMed PMID:23583636. 23583636 10.1016/j.jinf.2013.03.015 PMC7112694

[15] Public Health England, ISARIC (2013) Treatment of MERS-CoV: Decision Support Tool. Clinical Decision Making Tool for Treatment of MERS-CoV v.1.0, 18 June, 2013. Available at: http://www.hpa.org.uk/webc/HPAwebFile/HPAweb_C/1317139281416, Last Accessed 22 October 2013.

[16] de Wilde AH, Raj VS, Oudshoorn D, Bestebroer TM, van Nieuwkoop S, Limpens RW, Posthuma CC, van der Meer Y, Bárcena M, Haagmans BL, Snijder EJ, van den Hoogen BG. MERS-coronavirus replication induces severe in vitro cytopathology and is strongly inhibited by cyclosporin A or interferon-α treatment. J Gen Virol. 2013 Aug;94(Pt 8):1749-60. PubMed PMID:23620378. 23620378 10.1099/vir.0.052910-0 PMC3749523

[17] Josset L, Menachery VD, Gralinski LE, Agnihothram S, Sova P, Carter VS, Yount BL, Graham RL, Baric RS, Katze MG. Cell host response to infection with novel human coronavirus EMC predicts potential antivirals and important differences with SARS coronavirus. MBio. 2013 Apr 30;4(3):e00165-13. PubMed PMID:23631916. 23631916 10.1128/mBio.00165-13 PMC3663187

[18] Falzarano D, de Wit E, Martellaro C, Callison J, Munster VJ, Feldmann H. Inhibition of novel β coronavirus replication by a combination of interferon-α2b and ribavirin. Sci Rep. 2013;3:1686. PubMed PMID:23594967. 23594967 10.1038/srep01686 PMC3629412

[19] Mailles A, Blanckaert K, Chaud P, van der Werf S, Lina B, Caro V, Campese C, Guéry B, Prouvost H, Lemaire X, Paty MC, Haeghebaert S, Antoine D, Ettahar N, Noel H, Behillil S, Hendricx S, Manuguerra JC, Enouf V, La Ruche G, Semaille C, Coignard B, Lévy-Bruhl D, Weber F, Saura C, Che D. First cases of Middle East Respiratory Syndrome Coronavirus (MERS-CoV) infections in France, investigations and implications for the prevention of human-to-human transmission, France, May 2013. Euro Surveill. 2013 Jun 13;18(24). PubMed PMID:23787161. 23787161

[20] Puzelli S, Azzi A, Santini MG, Di Martino A, Facchini M, Castrucci MR, Meola M, Arvia R, Corcioli F, Pierucci F, Baretti S, Bartoloni A, Bartolozzi D, de Martino M, Galli L, Pompa MG, Rezza G, Balocchini E, Donatelli I. Investigation of an imported case of Middle East Respiratory Syndrome Coronavirus (MERS-CoV) infection in Florence, Italy, May to June 2013. Euro Surveill. 2013 Aug 22;18(34). PubMed PMID:23987829. 23987829 10.2807/1560-7917.es2013.18.34.20564

[21] WHO (2013) World Health Organization: MERS-CoV summary and literature update – as of 20 June 2013. Available at: http://www.who.int/csr/disease/coronavirus_infections/update_20130620/en/index.html. Last accessed 22 October 2013.

[22] Cauchemez S, Fraser C, Van Kerkhove MD, Donnelly CA, Riley S, et al. Middle East respiratory syndrome coronavirus: Quantifying the extent of the epidemic, surveillance biases and transmissibility thus far. The Lancet Infectious Diseases, Early Online Publication, 13 November 2013 doi:10.1016/S1473-3099(13)70304-9 10.1016/S1473-3099(13)70304-9 PMC3895322 24239323

[23] Buchholz U, Müller MA, Nitsche A, Sanewski A, Wevering N, Bauer-Balci T, Bonin F, Drosten C, Schweiger B, Wolff T, Muth D, Meyer B, Buda S, Krause G, Schaade L, Haas W. Contact investigation of a case of human novel coronavirus infection treated in a German hospital, October-November 2012. Euro Surveill. 2013 Feb 21;18(8). PubMed PMID:23449231. 23449231

[24] Pebody RG, Chand MA, Thomas HL, Green HK, Boddington NL, Carvalho C, Brown CS, Anderson SR, Rooney C, Crawley-Boevey E, Irwin DJ, Aarons E, Tong C, Newsholme W, Price N, Langrish C, Tucker D, Zhao H, Phin N, Crofts J, Bermingham A, Gilgunn-Jones E, Brown KE, Evans B, Catchpole M, Watson JM. The United Kingdom public health response to an imported laboratory confirmed case of a novel coronavirus in September 2012. Euro Surveill. 2012 Oct 4;17(40):20292. PubMed PMID:23078799. 23078799

[25] Memish ZA, Alhakeem R, Stephens GM. Saudi Arabia and the emergence of a novel coronavirus. East Mediterr Health J. 2013;19 Suppl 1:S7-11. PubMed PMID:23888789. 23888789

[26] Breban R, Riou J, Fontanet A. Interhuman transmissibility of Middle East respiratory syndrome coronavirus: estimation of pandemic risk. Lancet. 2013 Aug 24;382(9893):694-9. PubMed PMID:23831141. 23831141 10.1016/S0140-6736(13)61492-0 PMC7159280

[27] Cotten M, Watson SJ, Kellam P, Al-Rabeeah AA, Makhdoom HQ, Assiri A, Al-Tawfiq JA, Alhakeem RF, Madani H, Alrabiah FA, Hajjar SA, Al-Nassir WN, Albarrak A, Flemban H, Balkhy HH, Alsubaie S, Palser AL, Gall A, Bashford-Rogers R, Rambaut A, Zumla AI, Memish ZA. Transmission and evolution of the Middle East respiratory syndrome coronavirus in Saudi Arabia: a descriptive genomic study. Lancet. 2013 Sep 19. PubMed PMID:24055451. 24055451 10.1016/S0140-6736(13)61887-5 PMC3898949

[28] van Boheemen S, de Graaf M, Lauber C, Bestebroer TM, Raj VS, Zaki AM, Osterhaus AD, Haagmans BL, Gorbalenya AE, Snijder EJ, Fouchier RA. Genomic characterization of a newly discovered coronavirus associated with acute respiratory distress syndrome in humans. MBio. 2012 Nov 20;3(6). PubMed PMID:23170002. 23170002 10.1128/mBio.00473-12 PMC3509437

[29] Cotten M, Lam TT, Watson SJ, Palser AL, Petrova V, Grant P, Pybus OG, Rambaut A, Guan Y, Pillay D, Kellam P, Nastouli E. Full-genome deep sequencing and phylogenetic analysis of novel human betacoronavirus. Emerg Infect Dis. 2013 May;19(5):736-42B. PubMed PMID:23693015. 23693015 10.3201/eid1905.130057 PMC3647518

[30] Annan A, Baldwin HJ, Corman VM, Klose SM, Owusu M, Nkrumah EE, Badu EK, Anti P, Agbenyega O, Meyer B, Oppong S, Sarkodie YA, Kalko EK, Lina PH, Godlevska EV, Reusken C, Seebens A, Gloza-Rausch F, Vallo P, Tschapka M, Drosten C, Drexler JF. Human betacoronavirus 2c EMC/2012-related viruses in bats, Ghana and Europe. Emerg Infect Dis. 2013 Mar;19(3):456-9. PubMed PMID:23622767. 23622767 10.3201/eid1903.121503 PMC3647674

[31] Wacharapluesadee S, Sintunawa C, Kaewpom T, Khongnomnan K, Olival KJ, Epstein JH, Rodpan A, Sangsri P, Intarut N, Chindamporn A, Suksawa K, Hemachudha T. Group C betacoronavirus in bat guano fertilizer, Thailand. Emerg Infect Dis. 2013 Aug;19(8):1349-51. PubMed PMID:23880503. 23880503 10.3201/eid1908.130119 PMC3739538

[32] Anthony SJ, Ojeda-Flores R, Rico-Chávez O, Navarrete-Macias I, Zambrana-Torrelio CM, Rostal MK, Epstein JH, Tipps T, Liang E, Sanchez-Leon M, Sotomayor-Bonilla J, Aguirre AA, Ávila-Flores R, Medellín RA, Goldstein T, Suzán G, Daszak P, Lipkin WI. Coronaviruses in bats from Mexico. J Gen Virol. 2013 May;94(Pt 5):1028-38. PubMed PMID:23364191. 23364191 10.1099/vir.0.049759-0 PMC3709589

[33] Ithete NL, Stoffberg S, Corman VM, Cottontail VM, Richards LR, Schoeman MC, Drosten C, Drexler JF, Preiser W. Close relative of human middle East respiratory syndrome coronavirus in bat, South Africa. Emerg Infect Dis. 2013 Oct;19(10):1697-9. PubMed PMID:24050621. 24050621 10.3201/eid1910.130946 PMC3810765

[34] Memish ZA, Mishra N, Olival KJ, Fagbo SF, Kapoor V, Epstein JH, et al. Middle East respiratory syndrome coronavirus in bats, Saudi Arabia. Emerg Infect Dis [Internet]. 2013 Nov [date cited]. http://dx.doi.org/10.3201/eid1911.131172 10.3201/eid1911.131172 PMC3837665 24206838

[35] Reusken CB, Haagmans BL, Müller MA, Gutierrez C, Godeke GJ, Meyer B, Muth D, Raj VS, Vries LS, Corman VM, Drexler JF, Smits SL, El Tahir YE, De Sousa R, van Beek J, Nowotny N, van Maanen K, Hidalgo-Hermoso E, Bosch BJ, Rottier P, Osterhaus A, Gortázar-Schmidt C, Drosten C, Koopmans MP. Middle East respiratory syndrome coronavirus neutralising serum antibodies in dromedary camels: a comparative serological study. Lancet Infect Dis. 2013 Oct;13(10):859-66. PubMed PMID:23933067. 23933067 10.1016/S1473-3099(13)70164-6 PMC7106530

[36] Perera R, Wang P, Gomaa M, El-Shesheny R, Kandeil A, Bagato O, Siu L, Shehata M, Kayed A, Moatasim Y, Li M, Poon L, Guan Y, Webby R, Ali M, Peiris J, Kayali G. Seroepidemiology for MERS coronavirus using microneutralisation and pseudoparticle virus neutralisation assays reveal a high prevalence of antibody in dromedary camels in Egypt, June 2013. Euro Surveill. 2013 Sep 5;18(36). PubMed PMID:24079378. 24079378 10.2807/1560-7917.es2013.18.36.20574

[37] Corman VM, Eckerle I, Bleicker T, Zaki A, Landt O, Eschbach-Bludau M, van Boheemen S, Gopal R, Ballhause M, Bestebroer TM, Muth D, Müller MA, Drexler JF, Zambon M, Osterhaus AD, Fouchier RM, Drosten C. Detection of a novel human coronavirus by real-time reverse-transcription polymerase chain reaction. Euro Surveill. 2012 Sep 27;17(39). PubMed PMID:23041020. 23041020 10.2807/ese.17.39.20285-en

[38] United States Centers for Disease Control and Prevention (2013) Interim Guidelines for Collecting, Handling, and Testing Clinical Specimens from Patients Under Investigation (PUIs) for Middle East Respiratory Syndrome Coronavirus (MERS-CoV) – Version 2. Available at: http://www.cdc.gov/coronavirus/mers/guidelines-clinical-specimens.html. Last Accessed 22 October 2013.

[39] Corman VM, Müller MA, Costabel U, Timm J, Binger T, Meyer B, Kreher P, Lattwein E, Eschbach-Bludau M, Nitsche A, Bleicker T, Landt O, Schweiger B, Drexler JF, Osterhaus AD, Haagmans BL, Dittmer U, Bonin F, Wolff T, Drosten C. Assays for laboratory confirmation of novel human coronavirus (hCoV-EMC) infections. Euro Surveill. 2012 Dec 6;17(49). PubMed PMID:23231891. 23231891 10.2807/ese.17.49.20334-en

[40] Raj VS, Mou H, Smits SL, Dekkers DH, Müller MA, Dijkman R, Muth D, Demmers JA, Zaki A, Fouchier RA, Thiel V, Drosten C, Rottier PJ, Osterhaus AD, Bosch BJ, Haagmans BL. Dipeptidyl peptidase 4 is a functional receptor for the emerging human coronavirus-EMC. Nature. 2013 Mar 14;495(7440):251-4. PubMed PMID:23486063. 23486063 10.1038/nature12005 PMC7095326

[41] Lu G, Hu Y, Wang Q, Qi J, Gao F, Li Y, Zhang Y, Zhang W, Yuan Y, Bao J, Zhang B, Shi Y, Yan J, Gao GF. Molecular basis of binding between novel human coronavirus MERS-CoV and its receptor CD26. Nature. 2013 Aug 8;500(7461):227-31. PubMed PMID:23831647. 23831647 10.1038/nature12328 PMC7095341

[42] Müller MA, Raj VS, Muth D, Meyer B, Kallies S, Smits SL, Wollny R, Bestebroer TM, Specht S, Suliman T, Zimmermann K, Binger T, Eckerle I, Tschapka M, Zaki AM, Osterhaus AD, Fouchier RA, Haagmans BL, Drosten C. Human coronavirus EMC does not require the SARS-coronavirus receptor and maintains broad replicative capability in mammalian cell lines. MBio. 2012 Dec 11;3(6). PubMed PMID:23232719. 23232719 10.1128/mBio.00515-12 PMC3520110

[43] Chan JF, Chan KH, Choi GK, To KK, Tse H, Cai JP, Yeung ML, Cheng VC, Chen H, Che XY, Lau SK, Woo PC, Yuen KY. Differential cell line susceptibility to the emerging novel human betacoronavirus 2c EMC/2012: implications for disease pathogenesis and clinical manifestation. J Infect Dis. 2013 Jun 1;207(11):1743-52. PubMed PMID:23532101. 23532101 10.1093/infdis/jit123 PMC7107374

[44] Lambeir AM, Durinx C, Scharpé S, De Meester I. Dipeptidyl-peptidase IV from bench to bedside: an update on structural properties, functions, and clinical aspects of the enzyme DPP IV. Crit Rev Clin Lab Sci. 2003 Jun;40(3):209-94. PubMed PMID:12892317. 12892317 10.1080/713609354

[45] Gierer S, Bertram S, Kaup F, Wrensch F, Heurich A, Krämer-Kühl A, Welsch K, Winkler M, Meyer B, Drosten C, Dittmer U, von Hahn T, Simmons G, Hofmann H, Pöhlmann S. The spike protein of the emerging betacoronavirus EMC uses a novel coronavirus receptor for entry, can be activated by TMPRSS2, and is targeted by neutralizing antibodies. J Virol. 2013 May;87(10):5502-11. PubMed PMID:23468491. 23468491 10.1128/JVI.00128-13 PMC3648152

[46] Mou H, Raj VS, van Kuppeveld FJ, Rottier PJ, Haagmans BL, Bosch BJ. The receptor binding domain of the new Middle East respiratory syndrome coronavirus maps to a 231-residue region in the spike protein that efficiently elicits neutralizing antibodies. J Virol. 2013 Aug;87(16):9379-83. PubMed PMID:23785207. 23785207 10.1128/JVI.01277-13 PMC3754068

[47] Du L, Zhao G, Kou Z, Ma C, Sun S, Poon VK, Lu L, Wang L, Debnath AK, Zheng BJ, Zhou Y, Jiang S. Identification of a receptor-binding domain in the s protein of the novel human coronavirus Middle East respiratory syndrome coronavirus as an essential target for vaccine development. J Virol. 2013 Sep;87(17):9939-42. PubMed PMID:23824801. 23824801 10.1128/JVI.01048-13 PMC3754113

[48] Kindler E, Jónsdóttir HR, Muth D, Hamming OJ, Hartmann R, Rodriguez R, Geffers R, Fouchier RA, Drosten C, Müller MA, Dijkman R, Thiel V. Efficient replication of the novel human betacoronavirus EMC on primary human epithelium highlights its zoonotic potential. MBio. 2013 Feb 19;4(1):e00611-12. PubMed PMID:23422412. 23422412 10.1128/mBio.00611-12 PMC3573664

[49] Chan RW, Chan MC, Agnihothram S, Chan LL, Kuok DI, Fong JH, Guan Y, Poon LL, Baric RS, Nicholls JM, Peiris JS. Tropism of and innate immune responses to the novel human betacoronavirus lineage C virus in human ex vivo respiratory organ cultures. J Virol. 2013 Jun;87(12):6604-14. PubMed PMID:23552422. 23552422 10.1128/JVI.00009-13 PMC3676115

[50] Tao X, Hill TE, Morimoto C, Peters CJ, Ksiazek TG, Tseng CT. Bilateral entry and release of Middle East respiratory syndrome coronavirus induces profound apoptosis of human bronchial epithelial cells. J Virol. 2013 Sep;87(17):9953-8. PubMed PMID:23824802. 23824802 10.1128/JVI.01562-13 PMC3754134

[51] Kopecky-Bromberg SA, Martínez-Sobrido L, Frieman M, Baric RA, Palese P. Severe acute respiratory syndrome coronavirus open reading frame (ORF) 3b, ORF 6, and nucleocapsid proteins function as interferon antagonists. J Virol. 2007 Jan;81(2):548-57. PubMed PMID:17108024. 17108024 10.1128/JVI.01782-06 PMC1797484

[52] Reusken C, Mou H, Godeke GJ, van der Hoek L, Meyer B, Müller MA, Haagmans B, de Sousa R, Schuurman N, Dittmer U, Rottier P, Osterhaus A, Drosten C, Bosch BJ, Koopmans M. Specific serology for emerging human coronaviruses by protein microarray. Euro Surveill. 2013 Apr 4;18(14):20441. PubMed PMID:23594517. 23594517 10.2807/1560-7917.es2013.18.14.20441

[53] World Health Organization (2013) WHO guidelines for investigation of cases of human infection with Middle East Respiratory Syndrome Coronavirus (MERS-CoV) July 2013. Avaiable at: http://www.who.int/csr/disease/coronavirus_infections/MERS_CoV_investigation_guideline_Jul13.pdf. Last accessed 22 October 2013.

[54] Consortium for the Standardization of Influenza Seroepidemiology (CONSISE) (2013) CONSISE and Novel Coronavirus Resources. Available at: http://consise.tghn.org/articles/novel-coronavirus-ncov/. Last accessed 22 October 2013.

[55] Leung GM, Lim WW, Ho LM, Lam TH, Ghani AC, Donnelly CA, Fraser C, Riley S, Ferguson NM, Anderson RM, Hedley AJ. Seroprevalence of IgG antibodies to SARS-coronavirus in asymptomatic or subclinical population groups. Epidemiol Infect. 2006 Apr;134(2):211-21. PubMed PMID:16490123. 16490123 10.1017/S0950268805004826 PMC2870380

[56] Lee N, Allen Chan KC, Hui DS, Ng EK, Wu A, Chiu RW, Wong VW, Chan PK, Wong KT, Wong E, Cockram CS, Tam JS, Sung JJ, Lo YM. Effects of early corticosteroid treatment on plasma SARS-associated Coronavirus RNA concentrations in adult patients. J Clin Virol. 2004 Dec;31(4):304-9. PubMed PMID:15494274. 15494274 10.1016/j.jcv.2004.07.006 PMC7108318

[57] Brun-Buisson C, Richard JC, Mercat A, Thiébaut AC, Brochard L. Early corticosteroids in severe influenza A/H1N1 pneumonia and acute respiratory distress syndrome. Am J Respir Crit Care Med. 2011 May 1;183(9):1200-6. PubMed PMID:21471082. 21471082 10.1164/rccm.201101-0135OC

[58] Han K, Ma H, An X, Su Y, Chen J, Lian Z, Zhao J, Zhu BP, Fontaine RE, Feng Z, Zeng G. Early use of glucocorticoids was a risk factor for critical disease and death from pH1N1 infection. Clin Infect Dis. 2011 Aug;53(4):326-33. PubMed PMID:21810744. 21810744 10.1093/cid/cir398

[59] Kim SH, Hong SB, Yun SC, Choi WI, Ahn JJ, Lee YJ, Lee HB, Lim CM, Koh Y. Corticosteroid treatment in critically ill patients with pandemic influenza A/H1N1 2009 infection: analytic strategy using propensity scores. Am J Respir Crit Care Med. 2011 May 1;183(9):1207-14. PubMed PMID:21471084. 21471084 10.1164/rccm.201101-0110OC

[60] Martin-Loeches I, Lisboa T, Rhodes A, Moreno RP, Silva E, Sprung C, Chiche JD, Barahona D, Villabon M, Balasini C, Pearse RM, Matos R, Rello J. Use of early corticosteroid therapy on ICU admission in patients affected by severe pandemic (H1N1)v influenza A infection. Intensive Care Med. 2011 Feb;37(2):272-83. PubMed PMID:21107529. 21107529 10.1007/s00134-010-2078-z PMC7079858

[61] Dellinger RP, Levy MM, Rhodes A, Annane D, Gerlach H, Opal SM, Sevransky JE, Sprung CL, Douglas IS, Jaeschke R, Osborn TM, Nunnally ME, Townsend SR, Reinhart K, Kleinpell RM, Angus DC, Deutschman CS, Machado FR, Rubenfeld GD, Webb S, Beale RJ, Vincent JL, Moreno R. Surviving Sepsis Campaign: international guidelines for management of severe sepsis and septic shock, 2012. Intensive Care Med. 2013 Feb;39(2):165-228. PubMed PMID:23361625. 23361625 10.1007/s00134-012-2769-8 PMC7095153

[62] International Severe Acute Respiratory and Emerging Infection Consortium (ISARIC) (2013) ISARIC and WHO SARI and Natural History Protocols. Available at: http://isaric.tghn.org/articles/isaric-and-who-sari-and-natural-history-protocols/. Last accessed 22 October 2013.

[63] World Health Organization (2013) Infection prevention and control during health care for probable or confirmed cases of novel coronavirus (nCoV) infection. WHO, 2013 (http://www.who.int/csr/disease/coronavirus_infections/IPCnCoVguidance_06May13.pdf). Last Accessed 22 October 2013.

[64] Omrani AS, Matin MA, Haddad Q, Al-Nakhli D, Memish ZA, Albarrak AM. A family cluster of Middle East Respiratory Syndrome Coronavirus infections related to a likely unrecognized asymptomatic or mild case. Int J Infect Dis. 2013 Sep;17(9):e668-72. PubMed PMID:23916548. 23916548 10.1016/j.ijid.2013.07.001 PMC7110537

[65] Falzarano D, de Wit E, Rasmussen AL, Feldmann F, Okumura A, Scott DP, Brining D, Bushmaker T, Martellaro C, Baseler L, Benecke AG, Katze MG, Munster VJ, Feldmann H. Treatment with interferon-α2b and ribavirin improves outcome in MERS-CoV-infected rhesus macaques. Nat Med. 2013 Oct;19(10):1313-7. PubMed PMID:24013700. 24013700 10.1038/nm.3362 PMC4093902

